# Computational Modeling of Lipid Metabolism in Yeast

**DOI:** 10.3389/fmolb.2016.00057

**Published:** 2016-09-27

**Authors:** Vera Schützhold, Jens Hahn, Katja Tummler, Edda Klipp

**Affiliations:** Theoretical Biophysics, Institute for Biology, Humboldt-Universität zu BerlinBerlin, Germany

**Keywords:** lipidomics, *Saccharomyces cerevisiae*, Gillespie algorithm, spatial distribution of lipids, fatty acid saturation

## Abstract

Lipid metabolism is essential for all major cell functions and has recently gained increasing attention in research and health studies. However, mathematical modeling by means of classical approaches such as stoichiometric networks and ordinary differential equation systems has not yet provided satisfactory insights, due to the complexity of lipid metabolism characterized by many different species with only slight differences and by promiscuous multifunctional enzymes. Here, we present an object-oriented stochastic model approach as a way to cope with the complex lipid metabolic network. While all lipid species are treated objects in the model, they can be modified by the respective converting reactions based on reaction rules, a hybrid method that integrates benefits of agent-based and classical stochastic simulation. This approach allows to follow the dynamics of all lipid species with different fatty acids, different degrees of saturation and different headgroups over time and to analyze the effect of parameter changes, potential mutations in the catalyzing enzymes or provision of different precursors. Applied to yeast metabolism during one cell cycle period, we could analyze the distribution of all lipids to the various membranes in time-dependent manner. The presented approach allows to efficiently treat the complexity of cellular lipid metabolism and to derive conclusions on the time- and location-dependent distributions of lipid species and their properties such as saturation. It is widely applicable, easily extendable and will provide further insights in healthy and diseased states of cell metabolism.

## Introduction

### The importance of lipid metabolism in health and disease

Lipids are crucial players in a wide range of cellular processes. Their production is required for cell cycle progression and cell division. Furthermore, lipids are important mediators in signaling pathways, components of essential cofactors, building blocks of lipoproteins and glycerolipids and can be used to store and mobilize excess energy. In those contexts lipids execute structural as well as functional roles: By building membranes, they can create isolated reaction environments. The membranes form diffusion barriers and stabilize membrane associated enzymes (Daum et al., 1998). Lipids are also required to structurally anchor agents to specific regions of the cell, thereby substantially altering their activity. In form of cofactors, they actively take part in enzymatic reactions. Lipids can also be direct mediators of cellular signaling, for example in the regulation of endocytosis, ubiquitin dependent proteolysis or cell cycle control (Nielsen, 2009).

The internal lipid composition of membranes is variable and shapes membrane properties such as viscosity and permeability. For example, the sterol content defines the integrity of a membrane (Klug and Daum, 2014). Hence, lipid metabolism needs to supply the required membrane lipids in the right proportions and adapt them in response to external cues. In situations of excess energy supply, lipid metabolism can be rerouted to produce high energy, low maintenance storage compounds, which accumulate intracellularly in lipid droplets. Their dynamic build up and consumption ensures the energy-save passage through cell cycle.

This multitude of functions in turn also involves lipid metabolism in a wide range of pathological contexts. Due to the biosynthetic aspect, altered lipid metabolism has implications in mammalian cancer development (Natter and Kohlwein, 2013). In neurodegenerative diseases, such as Alzheimer's, accumulation of cholesterol in combination with the apolipoprotein E in the brain is thought to be a major risk factor (Liu et al., 2013) along with changes in the sphingolipid metabolism (van Echten-Deckert and Walter, 2012). An altered lipid metabolism has also been observed in kidney aging and related diseases (Braun et al., 2016). Increased lipogenesis occurs in obesity (Diraison et al., 2002), insulin resistance (Adams et al., 2005) and other metabolic diseases (Ameer et al., 2014).

Despite their essentiality, lipids only recently came into focus of systematic studies, aiming to understand the lipid contribution to a fully functional cell. One reason could be their extreme diversity that poses difficult challenges to modern -omics approaches. Mammalian cells contain thousands of different lipids (Fahy et al., 2009), with a multitude of potential functions, which are far from being understood in detail. In addition, the enzymes of lipid metabolic routes are often promiscuous, handling many different fatty acid species with appropriate efficiency. The resulting combinatorial complexity hampers an easy reconstruction of mass and information flow in the lipid processing system.

### Systems biology of lipid metabolism

The large number of involved reactions as well as the combinatorial complexity of the fatty acids, render studying lipid metabolism by means of classical molecular biology almost infeasible. Systems biology approaches can help to disentangle such complex systems and to keep track of the large numbers of involved entities. In addition, the integration and analysis of different complex, high-dimensional datasets as produced by novel -omics approaches can greatly contribute to the understanding of such large biological networks.

#### Experimental studies

In recent years, mass spectrometry based lipidomics have greatly advanced the understanding of cellular lipid biology. Experimental studies focused on describing the general nature of the lipidome, including lipid transfer, composition, diversity, and intracellular location in eukaryotic cells (Chiapparino et al., 2016) or the overall lipid composition of yeast cells (Ejsing et al., 2009). Effects of temperature, growth phase and media composition on the lipid composition have been measured (Klose et al., 2012), as well as screening studies of yeast mutant libraries to identify key genes (Tarasov et al., 2014). In plants, organellar lipidomics are well established (Horn and Chapman, 2011), but for yeast and mammalian cells, such data—dissecting growth or composition of subcellular membranes—is either lacking, or based on older sources (Zambrano et al., 1975; Griffiths et al., 1989; Zinser et al., 1991).

To handle the vast amount of data, standardization efforts have established a comprehensive classification system for lipids as an interface between experimental and computational approaches, allowing for the unique identification of lipids in experimental datasets (Lipid Maps, Fahy et al., 2009; van Meer and de Kroon, 2011). To analyze these kind of complex and interdependent datasets, mathematical modeling has proven to be a powerful tool.

#### Common modeling approaches for metabolic networks and lipid metabolism

Computational systems biology uses two major approaches to study metabolic networks. On the one hand, the distribution of metabolite fluxes can be described by flux balance analysis (FBA), a constraint based method that considers the stoichiometric wiring of reactions in a cell. FBA models often aim to cover the entirety of metabolic reactions within a cell. Extended methods can include thermodynamic, fluxomic, proteomic or gene expression data, but all lack the ability to capture dynamic changes in the system. On the other hand, small scale ordinary differential equation (ODE) based models simulate the time evolution of fluxes and metabolite concentrations, while integrating detailed knowledge about enzyme kinetics. They require a much deeper understanding of the examined system alongside a dense data coverage, but also yield in-depth, functional understanding.

Both methods are based on the detailed topological description of reactions and metabolites in the modeled network—which, in the case of lipid metabolism, is highly complex with the need to account for thousands of combinations of many enzymes with even more lipid species.

#### Stochastic modeling methods—the stochastic simulation algorithm and agent-based modeling

Opposed to the above described deterministic methods, several modeling approaches try to capture stochastic aspects of biological systems. Those become important at small molecule numbers that show a stochastic interaction with their environment and such that their time evolution cannot be described accurately by the mean over their population.

One common method is agent-based modeling, where the agents are autonomous objects that are able to make decisions. Following defined rules, each agent decides in each time step how to behave, depending on their own state as well as on interactions with other agents (Cilfone et al., 2015). Agent-based models are the highly flexible in the implementation of rules and can be applied to large, heterogeneous populations (Bonabeau, 2002), as they occur in lipid metabolism. Spatial aspects can be easily included to simulate for example the behavior of cell populations. They are less frequently used for biochemical networks, where the agents would be molecules and very numerous.

Also the stochastic simulation algorithm (SSA, Gillespie, 1976) tackles the stochastic nature of biochemical reactions. As opposed to the agent-centered approach, SSA simulates all successive reactions occurring in a system. For its execution, two random numbers are required for each reaction event: One defining the waiting time until the next reaction event, and one selecting which reaction is executed after that waiting time. The simulation will produce one time resolved realization of the chemical master equation of the modeled system. For a full understanding, the system is usually simulated many times, to relieve the bias of stochastic noise.

Especially for stiff systems (i.e., systems combining wildly different time scales) the classical stochastic simulation algorithm requires extensive computation times, as it simulates each reaction step successively. A workaround for such problems can be τ-leaping (Gillespie, 2001; Rathinam et al., 2003). In this case a fixed time interval τ is set, and all reactions occurring during this time in the stochastic system are executed at once, thereby drastically reducing the number of steps taken during one simulation. However, only for small τ-values we can assume that the reaction propensities remain constant during that interval, which results in a simulation error increasing with the size of τ.

These stochastic methods are dealing with many different types of molecules, which do not require one detailed reaction equation for each type. Rather, the algorithms keep track of each *molecule* in the system and allows it to be modified by a set of applicable reactions. These reactions can be defined per class of reactants (i.e., all phospholipids, regardless of their side chains, can be dephosphorylated), drastically reducing the complexity of the implementation.

Stochastic simulations are usually not applied to metabolic systems, due to the high concentrations of reactants, which would result in short time steps and long computation times. In the case of lipid metabolism, this might be true for the overall system (the concentration of lipids within the cell is high) but due to the vast diversity of lipid types, single species can easily be represented in a very low number only.

### Existing models of lipid metabolism

Modeling lipid metabolism, especially in the model system yeast, was mostly tackled by FBA based approaches. Initial genome scale models did, however, not cover the lipid metabolism in great detail. Nookaew et al. (2008), extended the existing model *iFF708* (Förster et al., 2003) specifically to include important reactions in lipid metabolism, thereby identifying new reporter metabolites indicating neighboring reactions responding to system perturbations. The model was further expanded, also with regard to the lipid metabolism, by the YeastNet community (e.g., Yeast 4.0, Dobson et al., 2010).

Dynamic pathway models are available for the sphingolipid pathway in yeast (Alvarez-Vasquez et al., 2004), along with its experimental validation (Alvarez-Vasquez et al., 2005). Fatty acid beta oxidation has been described recently by an ODE based model by van Eunen et al. (2013). The specific metabolism of lipoprotein particles was modeled by Knoblauch and co-workers to describe the dependency of serum lipid concentrations on various genetic and environmental cues (Knoblauch et al., 2000). Especially for cholesterol metabolism, with its many implications in cardiovascular diseases, a number of models on different levels of granularity have been developed (reviewed in Mc Auley and Mooney, 2015).

All of the above models do not provide a comprehensive view of lipid metabolism and its dynamics. They also neglect the spatial aspect of distributing lipids to their designated membranes and the establishment of specific lipid compositions within them. Here, we therefore present a model, which will use yeast as model organism and focus on a comprehensive description of lipid synthesis and distribution. This first application of stochastic, agent-based modeling to lipid metabolism will highlight the unique benefits of such approaches to resolve metabolic complexity and ambiguity. As in detailed ODE models, time evolutions and dependencies on substrates are covered, but without the requirement for a large number of deterministic reaction equations. What can be seen as a proof of principle here, is easily extendable to larger systems and other biological questions.

### Yeast lipid metabolism—a test case of moderate complexity

Compared to most mammalian cells, yeast (*Saccharomyces cerevisiae*) uses a rather small set of lipid species to build up its membranes. The four main fatty acids found in yeast lipids are palmitic acid (C16:0), palmitoleic acid (C16:1), stearic acid (C18:0), and oleic acid (C18:1) (Daum et al., 1998), representing ~90% of the cellular lipids. An additional major compound, cerotic acid (C26:0), which is required for the synthesis of sphingolipids (Smith and Lester, 1974), is the most abundant of the very long chain fatty acids that make up ~1% of the cellular fatty acids (Schneiter and Kohlwein, 1997). Each lipid can occur in every membrane, but depending on the membrane functions, the compositions differ: e.g., there is a high ergosterol content in the plasma membrane to maintain its rigidity and cardiolipin is overrepresented in the inner mitochondrial membrane stabilizing transmembrane proteins (Gomez and Robinson, 1999).

As opposed to most enzymes in central carbon metabolism, which use one substrate exclusively, enzymes in lipid metabolism are promiscuous and can act on a whole substrate class. Prominent examples are enzymes responsible for chemical changes in the phospholipid headgroups or enzymes like the triacylglycerol (TAG) lipase Tgl4 which can also function as a steryl ester hydrolase (Rajakumari and Daum, 2010). The agent-based approach can cover all substrate specificities without the need for individual reaction equations.

The limited set of actors renders yeast a practicable test case for novel methods and more generalist approaches to study lipid metabolism and membrane dynamics. The general structures of lipid metabolic processes, such as the promiscuity of the involved enzymes, are thereby conserved. We choose to model the underlying production process of lipid *de novo* synthesis, which establishes all cellular membranes and covers the overall material flow of lipids in the cell. The produced lipids and their distribution can then be the connection point for other models of lipid related biological processes. In addition, lipid metabolism is well conserved between yeast and mammalian cells (e.g., Weeks et al., 1997). The approaches and results of our model can hence be easily adapted to other species or disease related questions to lipid metabolism.

## Materials and methods

### Model topology

The model represents the lipid metabolism in yeast. As the complexity of a metabolic system in a cell is much too high to be reproduced in a new modeling approach, we focused on the main reactions of the lipid producing *de novo* pathway of a yeast cell. The chosen reactions illustrate the production of lipid molecules from small molecule precursors from the central carbon metabolism in short, but continuous and without gaps. The reaction network was designed to connect all the major metabolites relevant to lipid metabolism in the cell, while keeping a low level of complexity. The included lipid species are the main molecule classes that are found in yeast cell membranes (see also Section Results).

### Agent-based model setup

**Figure 1:**
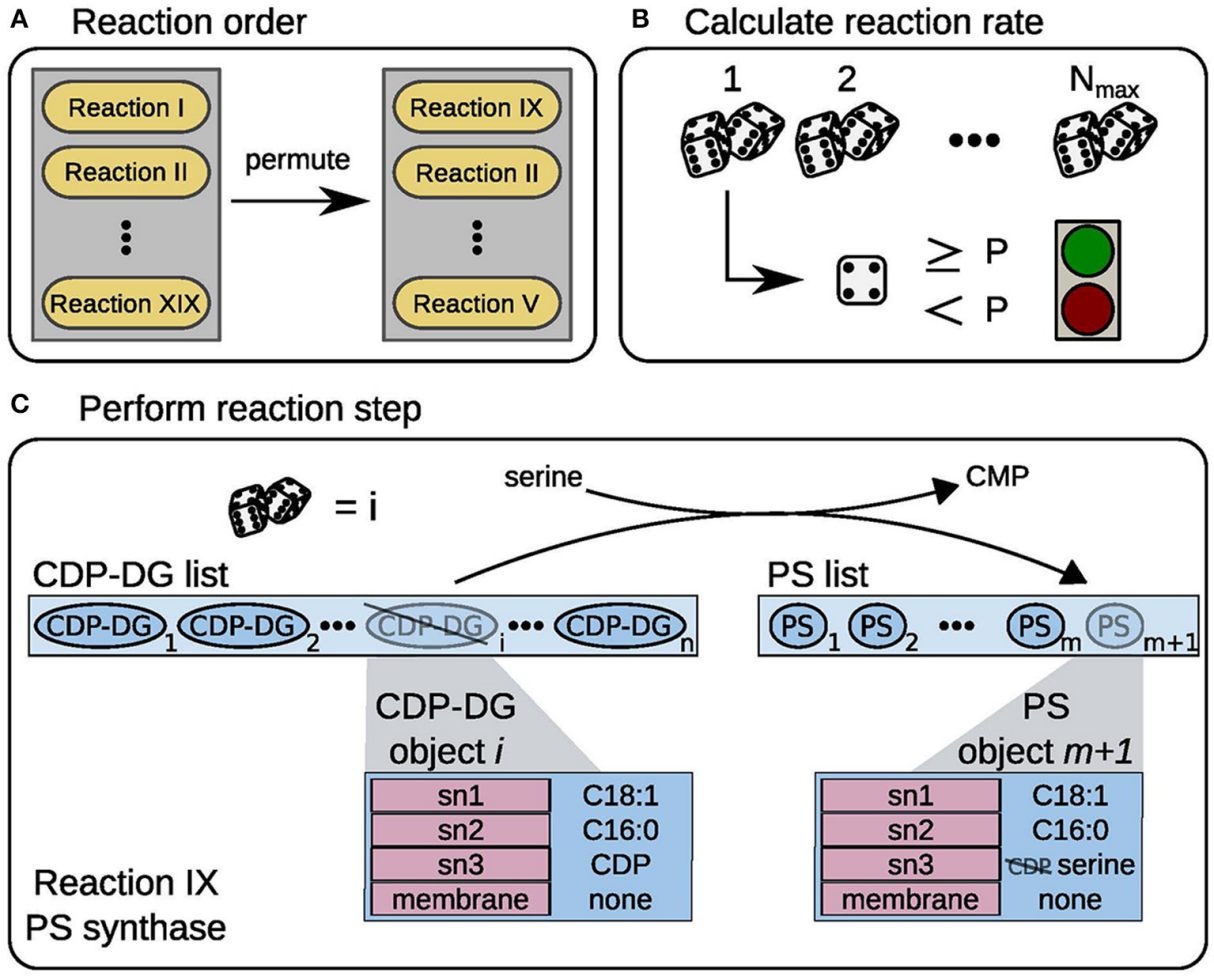
**Model workflow scheme**. Schematic representation of the model workflow, exemplified for PS synthase. **(A)** In every time step the order of reaction events is randomized. **(B)** A random number is drawn *N*_*max*_ times, if the number exceeds the threshold *P* the reaction is performed once. **(C)** A random CDP-DG object *i* is taken from the substrate list, here CDP-DG list. The reaction consumes a serine precursor molecule and releases a CMP from the CDP-DG headgroup (*sn3* position). The new PS object is appended to the product list, here PS list.

We implemented a simulation framework that combines aspects of agent-based modeling and τ-leaping in Python. We defined the participating biomolecules (*c.f.* Table 1) as objects, which can be equipped with a set of attributes. These attributes include bound elements (fatty acids with different saturation states or headgroups) and the localization to a specific membrane (*c.f.* Figure 1C). Reactions are implemented to modify those attributes, in dependency of the substrate availability and specific kinetic parameters. The reactions can thereby use different allowed substrates (i.e., fatty acids or headgroups) as defined by the reaction rules and append them to a specific lipid. We adopted the time discretization from τ-leaping: The model is simulated in 1 s time steps for 120 min, representing the transition through one cell cycle. Within this time interval, more than one reaction can be executed. For the stochastic simulation we use the random number generator from the Python package numpy, which implements a version of the Mersenne twister sequence. To save computation time, we scaled all compound numbers by a factor of 10^4^.

**Table 1:** **All lipid groups that are represented in the model with their category membership and headgroup**.

**Category**	**Name**	**Headgroup**	**Abbreviation**
Phospholipid	Phosphatidic acid	Phosphate	PA
	Phosphatidylserine	Serine	PS
	Phosphatidylethanolamine	Ethanolamine	PE
	Phosphatidylcholine	Choline	PC
	Phosphatidylinositol	Inositol	PI
	Cardiolipin	Phosphate	CL
Neutral lipid	Diacylglycerol	–	DAG
	Triacylglycerol	–	TAG
Sterol	Ergosterol	–	ES
	Steryl ester	–	SE
Sphingolipid	Mannosyl-diinositolphosphate ceramide	Inositol	SL

Each lipid group can form a several different species regarding to the attached fatty acids.

### Simulation of enzymatic reactions

**Figure 2:**
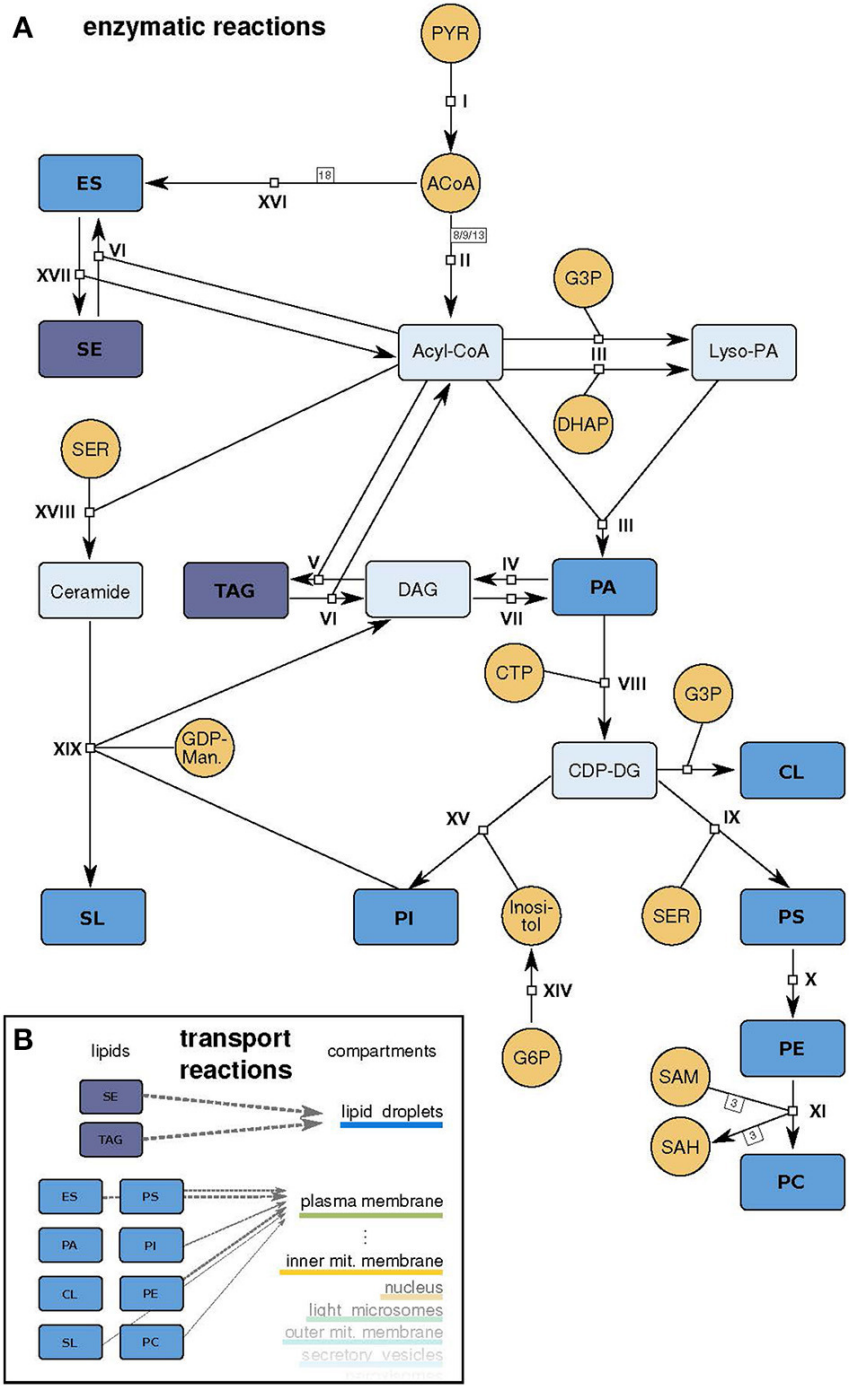
**Model scheme**. **(A)** Schematic representation of the model. Small molecule precursors, whose concentrations are held constant during the simulation, are depicted in orange, intermediate metabolites in light blue and lipids that can be incorporated in the different membranes in dark blue (abbreviations as in Table 1). The reactions *I - XIX* are described in detail in Supplementary Table 1. **(B)** Schematic representation of the transport reactions that move produced lipids to the compartment membranes with different stoichiometries (gray arrows).

To describe the production of lipids from metabolic precursors lumped enzymatic reactions were implemented, similarly to the rules in purely agent-based approaches (all reactions depicted in Figure 2A). We used two general parameters to describe how often each of those reactions is executed during the current time step. In analogy to the Michaelis-Menten kinetic, which is commonly used to describe enzymatic reactions, each reaction has a maximum number of executions *N*_*max*_ per time step (corresponding to the *v*_*max*_) and a certain probability *p* that the reaction actually takes place in each execution. Following the analogy, the probability is calculated from the substrate-limited *K*_*M*_ term in the MM equation as

(1) $$\begin{array}{l} p\;=\;\frac{\left[S\right]}{K_{M}\;+\;\left[S\right]} \end{array}$$

To implement substrate dependency, the probability *p* is updated before every time step according to the substrate concentrations after the previous time step. For reactions with more than one substrate we used a product of the above simple saturation terms for each substrate, resulting in exactly one *K*_*M*_ parameter per substrate and reaction.

Within one time step, the enzymatic reactions are then executed in the following manner: The order in which the reactions are executed is randomly permuted (Figure 1A). To calculate how many times reaction *i* in the ordered list is executed in the time step, we draw *N*_*max, i*_ random numbers. Each time the random number exceeds the current *p*_*i*_, we allow reaction *i* to run (Figure 1B). Each executed reaction changes the attributes of a lipid based on its rule (Figure 1C, one function for each reaction type, *c.f.* Supplementary Table 1). After the reactions took place, the precursor metabolite pools are replenished with given constant rates, simulating a flux from the central carbon metabolism.

### Simulation of transport reactions distributing produced lipids to membranes

The produced lipids are distributed with fixed, data based ratios to the different membranes via dedicated transport reactions (Figure 2B). The transport process is a pure agent-based implementation. Each lipid type has a certain probability to be localized to each membrane. The probabilities of the transport rules are thereby products of the membrane sizes (partially based on Uchida et al., 2011, see below) and the particular membrane composition based on the data of Zinser et al. (1991; *c.f.* Supplementary Table 2). The transport reaction distributes 10% of each lipid type to the membranes per time step by changing their “membrane” attribute. The transport reaction responsible for formation and degradation of lipid droplets are modeled via constant fluxes, which are dependent on the cell cycle phase (for the values see Supplementary Table 1).

### Model initialization

A start function is used to initialize the model, producing all membranes and free lipids required for a cell in early G1 phase, the first phase of the cell cycle. The amounts are adjusted to the data from Uchida et al. (2011), and the lipid compositions to the Zinser measurements (Zinser et al., 1991). Some noise is added, such that each simulation starts with a slightly different cell. Small molecule precursors are initialized before the simulation starts.

### Data basis and parameter determination

Kinetic parameters were adjusted manually to ensure that the model runs lead to results as close as possible to the given data from Zinser et al. (1991) and Uchida et al. (2011). Also, these data were used directly as probability factors for lipid transport (Supplementary Table 2).

For parameter adjustment, the model was executed many times. After each of these test model runs, the parameters *N*_*max*_ and *p* were manually changed stepwise to increase the accuracy of the reproduction of the given data. Therefore, the changes became smaller with higher numbers of test runs leading to the best precision possible. Finally, the maximum velocity *N*_*max*_ was highest for precursor molecules producing reactions (e.g., *N*_*max*(*acetyl*−*CoA synthase*)_ = 650) and low for lipid synthesis reactions (e.g., *N*_*max*(*PC synthase*)_ = 5). The production rate of precursor molecules was determined to not be the limiting factor for lipid synthesis leading to a nearly constant amount of them.

The study of Uchida et al. (2011) provides us with membrane surface areas of the nuclear membrane (12 μm^2^), the vacuolar membrane (5 μm^2^), and the outer mitochondrial membrane (5 μm^2^). The areas of the remaining subcellular membranes were unknown and set to physiologically reasonable estimates (Table 2). Uchida et al. (2011) also provides values for the average volume of the cell (50 fL), which we translate, assuming an ideal spherical cell to an initial area of the plasma membrane of 65.9 μm^2^. We assume that the cell doubles its size within the simulation time of 120 min (average period of one cell cycle) to 100 fL of which we account 60% (62.5 fL) to mother cell, the rest to the bud (37.5 fL). The assumption of two individual round cells leads to a total final plasma membrane surface of 130.4 μm^2^. Assuming a general conversion constant of 5 × 10^6^ lipids per μm^2^ lipid bilayer (Alberts et al., 2007) enables the calculation of lipid numbers per membrane, the unit used in the model simulations.

**Table 2:** **Membrane sizes at the start and the end of one cell cycle**.

**Membrane**	**Initial lipid number (model/data) × 10^4^**	**Final lipid number (data) × 10^4^**	**Mean final lipid number (model) × 10^4^**	**std of final lipid number (model) × 10^4^**
Plasma membrane	32950^*^	65900	60195	512
Secretory vesicles	500	1000	975	23
Vacuoles	2500^*^	12000^*^	10812	119
Nucleus	6000^*^	12500^*^	10756	93
Peroxisomes	500	1000	858	20
Light microsomes	500	1000	817	19
Inner mitochondrial membrane	5000^**^	7000^**^	6828	52
Outer mitochondrial membrane	2500^*^	3500^*^	3363	32
Lipid particles	1000	2000	1799	575

All Simulations are initialized with equally large membranes with noisy lipid distributions.

* Values calculated from Uchida et al. (2011),

** Calculated as twice the area of the outer mitochondrial membrane. All other final lipid numbers were assumed to be twice the initial number, unknown initial lipid numbers were assumed. Standard deviation (SD) and mean are calculated from 1000 model runs.

The distribution of fatty acids within the lipid species were based on experimental work from Martin et al. (2007). They state percentages of fatty acids in the pool of lipids of approximately 50% C18:1, 30% C16:1, 9% C16:0 and a small amount of C18:0. In the model, to reduce the number of possible fatty acids, we lump all remaining compounds that occur rarely into the C18:0 fatty acid species making up 11% altogether.

### Model output and availability

The model produces time series of the membrane lengths, which can be saved in table format. Also the final lipid composition and the fatty acid distribution can be requested.

All scripts for the model and simulations are made publicly available via GitHub https://github.com/tbphu/lipid_metabolism.

## Results

### Tackling combinatorial complexity

To solve the problem of combinatorial expansion and to allow time-dependent simulation of lipid metabolism in normal and perturbed states, we developed a hybrid object-oriented approach that combines aspects from agent-based modeling and generic stochastic simulation via the Gillespie algorithm. From the agent-based approach we adopt the definition of lipids as autonomous agents, implemented as Python objects. The lipid objects can be equipped with several attributes representing side chains, headgroups and membrane association, which are targets of modification by the lipid metabolic reactions. As the reactions “take the decision” about the fate of the lipid agents, not the agents themselves, the classical agent-based method is not applicable. We hence decided on an implementation close to the principles of Gillespie's Stochastic Simulation Algorithm (Gillespie, 1976), where the execution of a reaction step depends on two random numbers selecting the next executable reaction and its waiting time. Due to the object nature of the substrates, the reactions can be promiscuous, choosing with defined probabilities from a set of allowed substrates. This drastically reduces the complexity of the model setup, as we do not need to define all possible combinations of substrates and enzymes as individual reactions.

As another novel feature, we transfer the deterministic idea of the Michaelis-Menten kinetics to a stochastic environment, by changing the reaction propensities according to the current number of eligible substrate objects (see Section Materials and Methods and Supplementary Figure 1).

The implementation is described in more detail in the Materials and Methods section.

### Network topology and coverage

We apply our approach to the mayor lipid metabolic routes in yeast *S. cerevisiae.* The model includes 19 reactions of the lipid metabolism, whereby some are lumping several reaction steps of the living cell (Figure 2 and Supplementary Table 1). The modeled metabolism starts with small molecule precursors from the central carbon metabolism (pyruvate, dihydroxyacetone phosphate (DHAP), glycerol 3-phosphate) and ends with the newly synthesized lipids.

The model includes several subtypes of phospholipids, neutral lipids, sterols and a sphingolipid species (summarized in Table 1). The phospholipids phosphatidic acid (PA), phosphatidylserine (PS), phosphatidylethanolamine (PE), phosphatidylcholine (PC), and phosphatidylinositol (PI) contain two fatty acids at positions *sn1* and *sn2* and differ in their headgroup connected to position *sn3*, which determines the lipid class. Possible headgroups accounted for in the model are phosphate, cytidine diphosphate (CDP), serine, ethanolamine, choline, and inositol.

The main pathway in the lipid metabolism is the production of these phospholipids. All lipid species of this class are derived from PA. To generate this precursor lipid, which is also used as a membrane lipid itself, two fatty acids need to be added to a glycerol backbone. This backbone can be derived from glycerol 3-phosphate or a DHAP, two intermediates of the central carbon metabolism. The attachment of the nucleotide cytidine triphosphate (CTP) activates the PA headgroup, making it accessible for chemical changes: transformations to all other phospholipid species are now possible. Directly derived from the CDP-DG (active PA) are PI, cardiolipin (CL) and PS. PS in turn is used as a precursor for PE and which can be further processed to PC (Daum et al., 1998; Henry et al., 2012; Klug and Daum, 2014).

The model also includes lipids with more complex structures, with different numbers of attached fatty acids (triacylglycerides (TAG)), aromatic elements (sterols: ergosterol (ES) and steryl ester (SE)) or a ceramide backbone (sphingolipids (SL)). Ergosterol and the sphingolipids are synthesized via individual pathways with connections to intermediates of the phospholipid metabolism. The ergosterol synthesis in yeast normally includes more than 15 reactions and almost 30 enzymes, but is lumped to a single reaction in the model: 18 acetyl-CoA molecules build up one ergosterol (*c.f.* reaction XVI, Supplementary Table 1 and Figure 2). The sphingolipid synthesis reaction uses upstream intermediates (ceramide, PI) to produce the most abundant sphingolipid in yeast, mannosyl-diinositolphosphate ceramide, again as a lumped version of the entire synthesis route.

We include fatty acids of type C16:0, C16:1, C18:0, C18:1 as well as C26:0 for the synthesis of sphingolipids. Each lipid can have an individual combination of attached fatty acids, with the constraint of having an unsaturated fatty acid at the *sn2* position (Daum et al., 1998).

The boundary conditions for simulations of the lipid metabolism with our model are the following: Concentrations of small precursor molecules were considered constant, implemented by a refill reaction in each time step of the simulation. Their consumption was tracked during the simulation to ensure carbon conservation. The produced lipids are distributed to the respective membranes via transport reactions. These serve as a sink for free lipids and feed the growth of the individual membranes.

The yeast genome also encodes a functional Kennedy pathway for the production of PE and PC from extracellular precursors (Gibellini and Smith, 2010). In the model we do not include this scavenging pathway, as it would be difficult to assess it experimentally. The *de novo* synthesis, in contrast, has a defined input from the central carbon metabolism and is hence a clean experimental target that allows for easy validation.

### Data used for model calibration

#### Composition and size of the different cellular membranes

Despite the broad availability of novel lipidomics data, there are only few studies intending to resolve the distribution of lipids in the different subcellular membranes or their evolution during the different cell cycle phases. As we need this level of detail to calibrate the model, we used an older dataset from Zinser et al. (1991), who report measurements of the phospholipid compositions and the ergosterol to phospholipid ratio of subcellular fractions. Our developed lipid metabolism model is geared to these data and thus, exclusively the membranes characterized in Zinser et al. (1991) are used as membranes in the model: the plasma membrane, secretory vesicles' membranes, vacuolar, nuclear, peroxisomal, inner and outer mitochondrial membrane. The ergosterol to phospholipid ratio of 3.31 for the plasma membrane described by Zinser et al. (1991) is not used, because the biological plausibility is questioned by van der Rest et al. (1995) based on data by Patton and Lester (1991). Van der Rest et al. argue that the rigidity of a membrane with three sterols around each phospholipid would be too high to build a functional membrane and suggest a more lifelike value of 0.94 for the ratio, which we use as target value in the model.

A study by Uchida et al. (2011) measured the sizes and surface areas of yeast organelles by soft X-ray chromatography in different cell cycle phases. We used this data to define the sizes (i.e., number of lipids) in the nucleus, vacuole and outer mitochondrial membrane and used size estimates for the remaining membranes. A detailed description of how the target membrane sizes and compositions were obtained can be found in the Material and Methods section.

#### Behavior of lipid droplets

We additionally considered the dynamic build-up and exploitation of lipid droplets during the cell cycle. The cell cycle is divided into four phases, whereas two phases are gap times (G): the G1 phase, the synthesis phase (S phase), the G2 phase and at last the Mitosis, when cell division takes place. Lipid droplets store neutral lipids (TAG) and steryl esters during G1 phase and release them via a Cdk1/Cdc28-dependent activation of the lipase Tgl4 (Kurat et al., 2009) from S-phase on, when more lipid precursors are needed to build up new membranes for the growing bud. While lipid droplets have a multitude of functions and interactions (Wang, 2015), we only focus on their storage capacity in the model. We neglect the surrounding phospholipid monolayer and assume the droplets to consist only of TAG and steryl esters in a 50:50 ratio (Clausen et al., 1974). As our primary data source form Uchida et al. (2011) does not state size or volume of lipid particles, we assumed them to contain 10^7^ lipids at the start of G1 phase.

### Dynamic model behavior

#### Reproducing membrane growth and subcellular membrane composition

**Figure 3:**
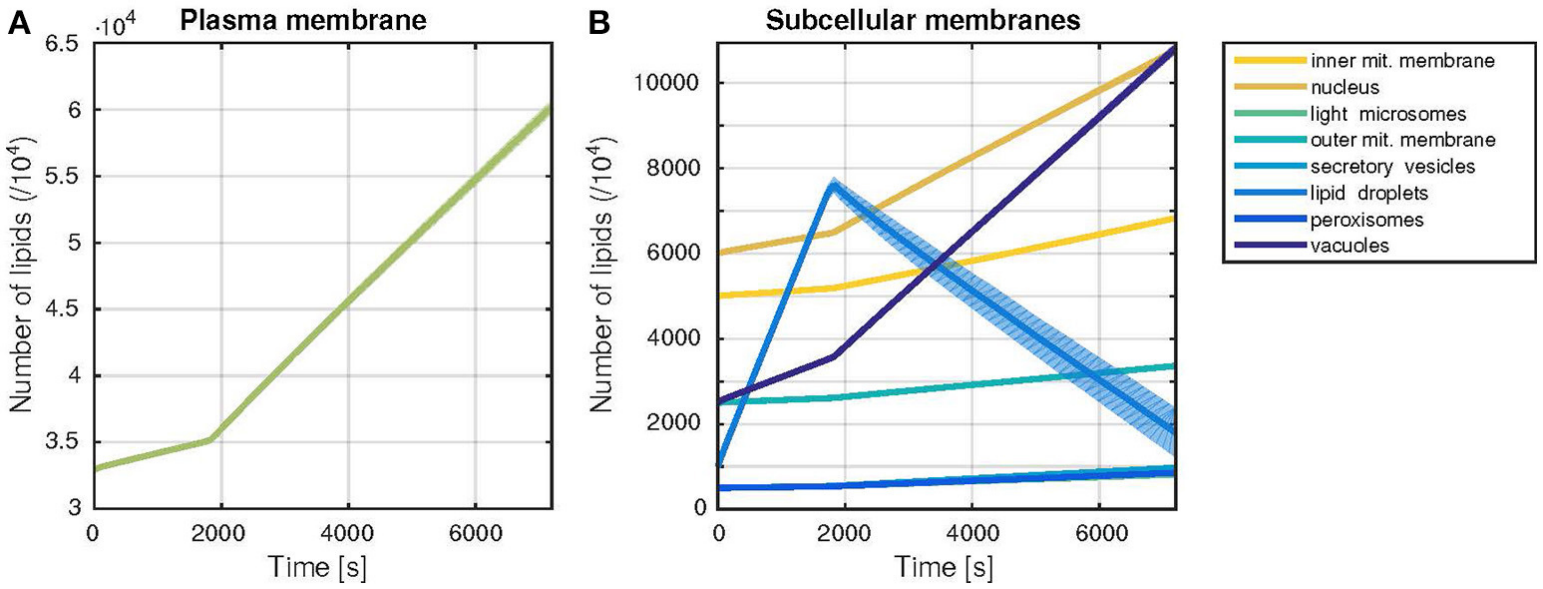
**Trajectories of the membrane sizes (time courses)**. Dynamic behavior of the model during one cell cycle, with a G1-phase duration of 30 min. The time evolution of the membrane sizes, measured by the number of lipids in each membrane is shown. Thick lines represent the mean, shaded areas the standard deviation of 1000 model simulations. **(A)** Plasma membrane growth **(B)** Growth of all other subcellular membranes occurring in the model.

The developed model was able to simulate the growth of all membranes over the time of one cell cycle (Figure 3). The simulated growth reproduces with satisfactory accuracy the benchmark data values (Table 2), while including stochastic effects. It also captures the dynamic build-up of lipid droplets during G1 phase (first 30 min of the simulation). Upon entry to S phase, the formation of the bud requires a higher biosynthetic capacity. In the model, the lipid droplets are consumed in this phase, to allow for a faster growth of all other organelles' membranes, in accordance with their behavior *in vivo* (Wang, 2015). The lipid droplets are also the component with the highest variability in its size (*c.f.* standard deviation in Figure 3B), which can be explained by the reversible nature of their build-up. In contrast to the remaining membranes, from which lipids cannot be freed once they were included in the membrane, neutral lipids can be released again from lipid droplets via the TAG lipase reaction.

**Figure 4:**
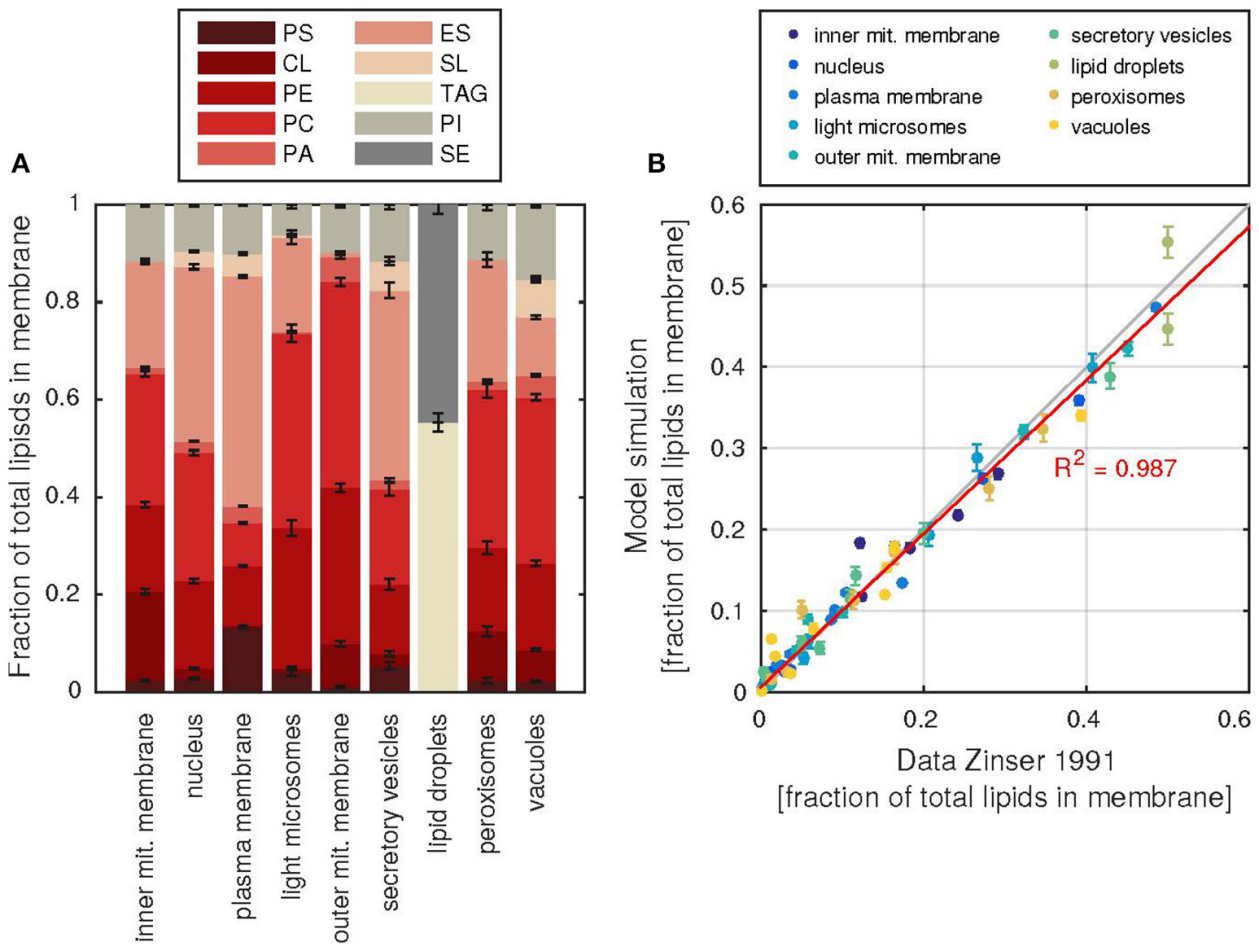
**Subcellular membrane composition and agreement with data. (A)** The relative contributions of each lipid class (abbreviations as in Table 1) to the total number of lipids in each membrane. Error bars denote the standard deviation of 1000 model simulations. **(B)** Agreement of the simulate fractions with the data of Zinser et al. (1991), each dot represents the fraction of a lipid in a subcellular membrane, all fractions of one membrane are colored according to the color key above. The red line describes a linear regression line between model and data fractions, the resulting coefficient of determination R^2^ is shown. Error bars as in **(A)**.

The lipid composition of each membrane can be retrieved from the model (Figure 4A and Supplementary Table 3). The membranes can have vastly different compositions, as was shown by Zinser et al. (1991), which is captured well by the model (Figure 4B). In addition, the model can also be used to gain insight into the fatty acid composition of the lipids, which again reproduces the experimental data of Martin and co-workers (Martin et al., 2007; Supplementary Figure 2).

#### Sensitivity analysis

Due to the model structure that combines the benefits of agent-based modeling with the classical approaches of enzyme kinetics, we can test and quantify the influence of parameter variations on the size and composition of the cellular membranes. For example, just like a common experiment in experimental biology, we can test the influence of changes in enzyme concentrations by modifying the *N*_*max*_ parameter, the equivalent to the Michaelis-Menten *v*_*max*_, the product of enzyme amount and catalytic activity.

**Figure 5:**
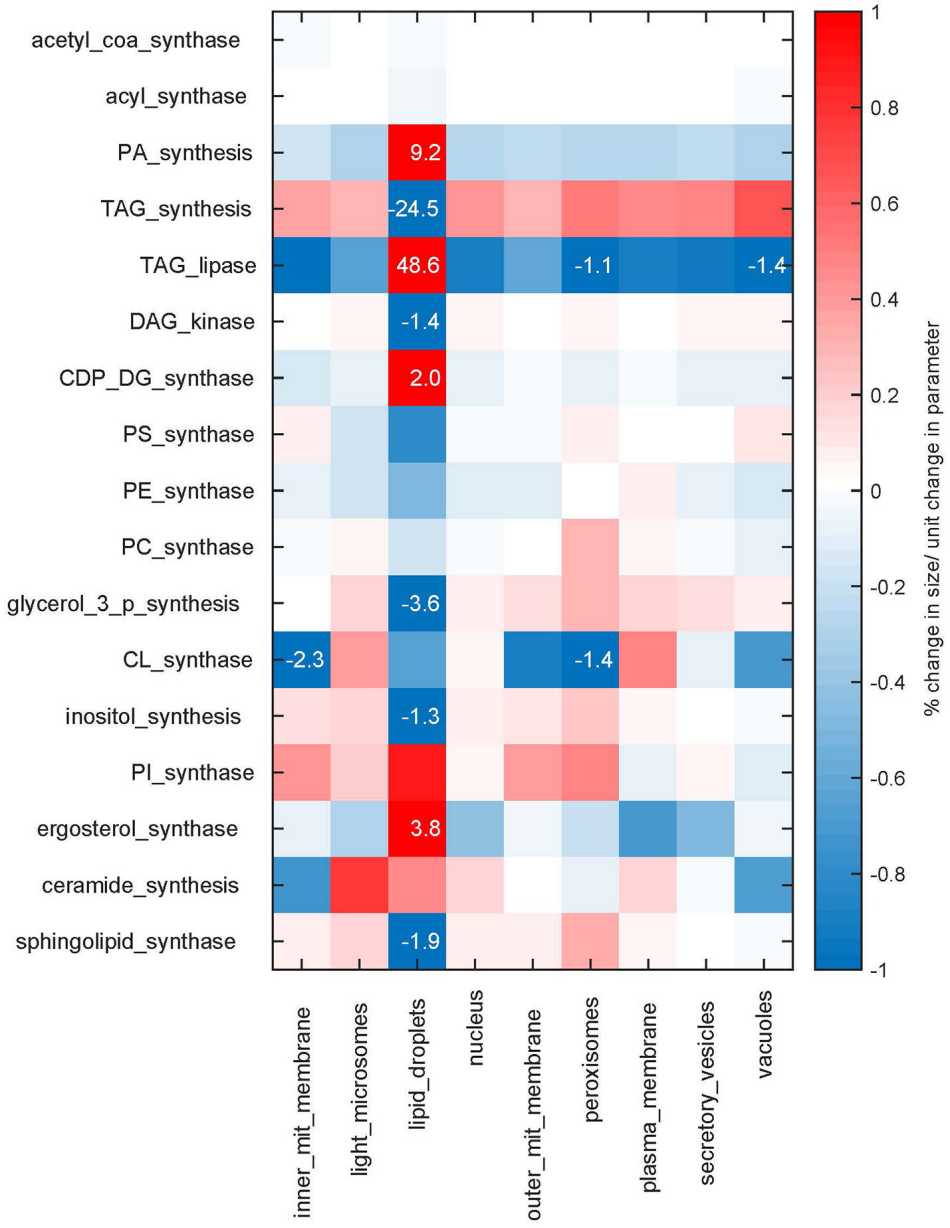
**Sensitivities**. Sensitivities of the membrane sizes to changes in the maximum rates *N*_*max*_ of the synthesis reactions. The sensitivities are shown as percent of change in membrane size upon a parameter change of one unit. Sensitivities are color coded, values larger than one percent are additionally given as numbers.

We see that in our modeled system, the size of the lipid droplets is most sensitive to perturbations in the reaction rates (Figure 5). In turn, the reactions which synthesize and degrade lipids from the droplets have a larger influence also on the sizes of the other cellular membranes, highlighting the importance of storage lipids and their mobilization. Overall the sensitivities are relatively small, with a maximum of 48.6% change per parameter unit changed (TAG lipase on lipid droplets), but with the majority of the sensitivities lying below 1% (see also Supplementary Figure 3 for sensitivities of membrane compositions). Hence, the model behavior is in general rather robust to changes in the rate parameters.

### Test cases of disturbed lipid metabolism

To assess the plausibility of the model, we simulated two biological test cases of disturbed lipid metabolism. In our implementation, all model parameters are easily accessible and can be modified to simulate experimental perturbations. We tested a case of altered enzyme activity as well as a perturbation in precursor availability as two structurally different experimental setups.

#### Inhibition of ergosterol synthesis

Leber et al. (1995) describe the effect of terbinafine treatment on the ergosterol content of the membranes. Terbinafine inhibits the ergosterol synthesis pathway such that no ergosterol can be produced by the cell. It does not affect the production of any other lipid species, resulting in growing membranes that are depleted of ergosterol. Leber et al. (1995) report a reduction of about 40% of the cholesterol content in comparison with untreated cells after 4 h of treatment.

**Figure 6:**
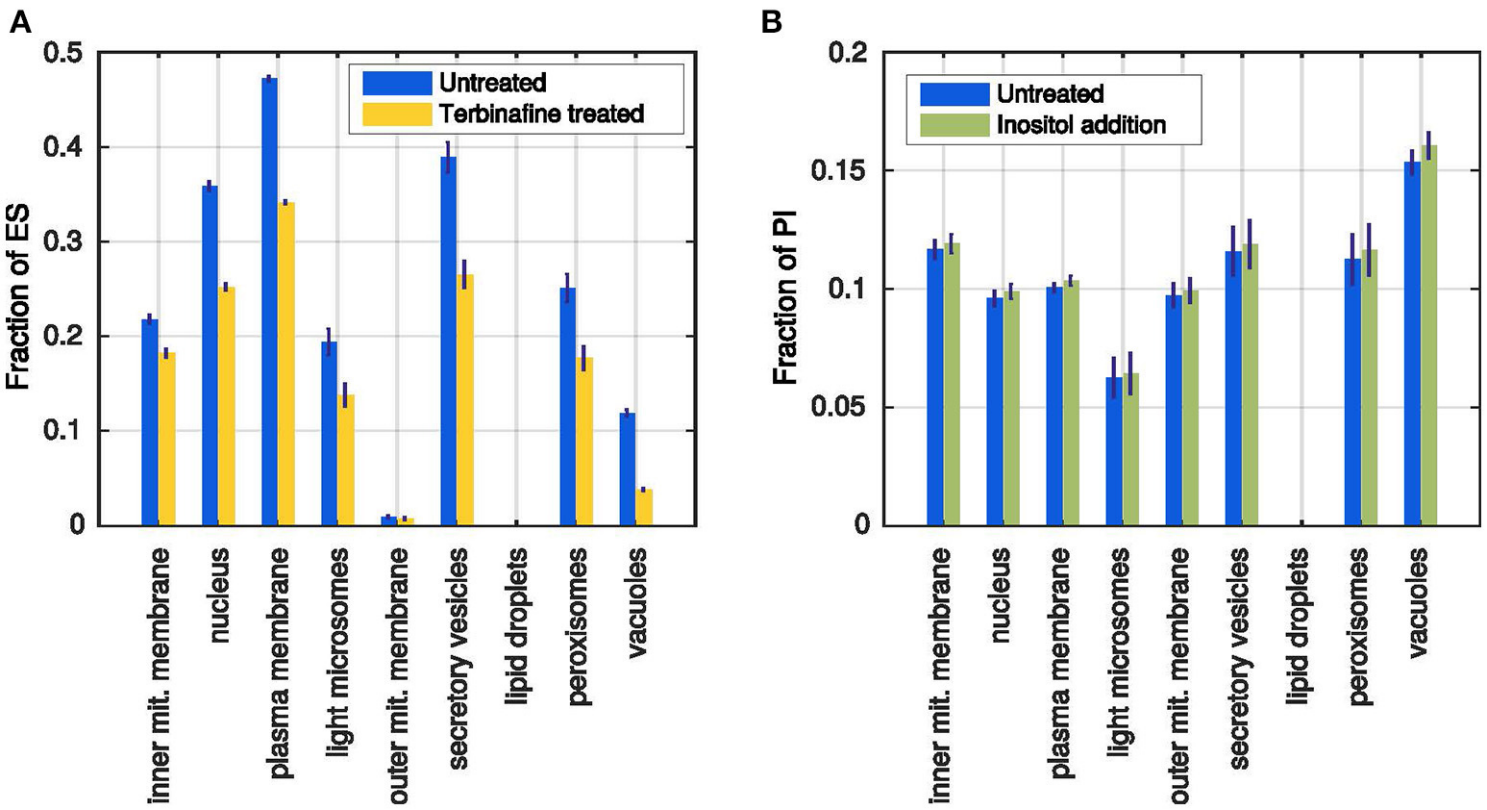
**Simulated test cases of the model behavior. (A)** Ergosterol content in each membrane after simulation of the terbinafine treatment (yellow) in comparison with previous untreated simulations (blue). **(B)** Fractions of PI in all membranes after standard simulation (blue) and simulated inositol addition (green). Error bars represent standard deviations of 1000 simulations.

In the model the treatment was simulated by setting the ergosterol production rate to 0, leading to an average reduction in ergosterol content of about 30% in all membranes compared to the unperturbed simulations (Figure 6A). Leber et al. (1995) also reports a slightly reduced cell growth as a side effect of the blocked ergosterol production, which we also observe in the simulated membrane growth (~80,000 instead of ~96,000 lipids total). The different membrane growth rates after ergosterol synthesis inhibition are also a proof for the utilization of the storage lipids: While lipids from the lipid droplets can be mobilized, all membranes grow faster. However, once the lipid droplets are completely emptied, the membranes grow with significantly decreased rates (Supplementary Figures 4A,B).

#### Addition of inositol in external medium

Loewen et al. (2004) reported that addition of inositol to the culture medium generally increases the content of PI. Inositol is a precursor for the synthesis of PI and as the reaction probabilities are dependent on substrate availabilities, the production of PI should be increased when more inositol is added to the model. We implemented the inositol addition as an additional refilling flux for the inositol precursor pool. In the unperturbed simulation, all inositol molecules needed to be produced from glucose 6-phosphate, whereas now they can be replenished directly from the external medium.

The resulting membrane compositions show that indeed there is a positive effect of the inositol addition to the PI production rate: All membranes have higher PI percentages at the end of the simulation time (Figure 6B), whereas the membrane growth is not affected (Supplementary Figures 4C,D). However, the reported massive increase in the overall PI formation, could not be reached in the simulation, where the largest effect was an about 5% higher PI content (vacuolar membrane). In conclusion, the model can reproduce the qualitative, but not the quantitative response of the cell to increased inositol availability. This can be due to specific parameter choices or due to the availability of precursors or due to the level of detail with respect to the included reactions and their regulation.

### Discussion

In this study we present a novel methodology for dynamic quantitative modeling of lipid metabolism. We cope with its inherent complexity by applying a hybrid approach combining the general concept of agent-based modeling with the temporal update strategies of the stochastic Gillespie algorithm. This integrative approach allows us to describe all lipid species and their evolution in time. We keep track of the main properties of all lipids such as headgroups, fatty acid lengths and saturation degrees as well as their localization. Applied to the comparatively simple lipid metabolism of the model organism Baker's yeast, we could demonstrate the temporal evolution of the lipid species in different cellular membranes. We could also successfully test different experimentally studied scenarios, such as addition of inositol to the medium or deficiency in ergosterol synthase.

The presented approach has a number of advantages and shortcoming when compared to the traditional approach in metabolism, i.e., the description of the dynamics of all components with ordinary differential equations, where all reactions are assigned to specific enzymes.

### Advantages

First of all, the mathematical complexity in our approach is computationally feasible. Since we avoid formulating differential equations for all individual potentially occurring lipid species, we prevent dealing with combinatorial explosion caused by different potential combinations of headgroups, fatty acid lengths and saturation degrees, even more complicated by the fact that many enzymes have multiple substrates. Instead, we use an object-oriented approach, where a lipid is an object that has rules stating which transformation it can undergo with defined probabilities. Promiscuity of enzymes can be addressed by defining individual substrate classes for reactions dependent on object properties. The flexibility of the object definitions provides furthermore an useful interface to integrate ambiguous data sets. This way of modeling will become especially helpful for more extended models considering even more types of (longer) fatty acids and additional saturation degrees.

Second, despite its structural difference from common metabolic modeling approaches, our implementation also allows to simulate many types of experimental perturbations (varying enzyme amounts, knockouts, enzyme activity changes, substrate concentration changes etc.). This can be used to understand the effects of single nodes in the lipid metabolic network in mechanistic detail, even if they should be inaccessible to experimental methods. Thus, comparable analysis opportunities are available as for common ODE based models.

### Limitations

While the approach allows in principle to keep track of every individual molecule, the molecule numbers in lipid metabolism are too high to allow for useful simulation times. Therefore, we applied an upscaling by multiplication with a factor (10^4^). In strict terms, this reduces the precision, however to a degree of no relevance. It does not seem to affect predictive power of the approach.

Hitherto, we have tested the approach. It can be extended by including further details of lipid metabolism such as (i) the transport of lipids between different membranes, (ii) lipid degradation and recycling, (iii) the influence of energetic or redox limitations in the cell on the model reactions or (iv) the mechanistic details of lipid distribution to the different membranes. The consideration of these aspects would allow for more detailed analyses, given that significant data become available to compare to.

In mathematical terms, the combination of agent-based modeling with stochastic simulation meets the general problem that there are no established methods for parameter estimation in stochastic systems. The aim is always a minimal deviation of the simulation results from available experimental data. While mean values are easy to obtain, it remains a challenge to compare higher moments. Here, we estimated two parameters for each reaction, the probability and the maximal velocity. A reaction and substrate specific Michaelis-Menten constant is calculated with this adjusted set of parameters at the beginning of a model run and is used for all executions of this particular reaction. To test the validity of the approach, we compared the model's stochastic description with the deterministic Michaelis-Menten-kinetic. As shown in Supplementary Figure 1, when using the same parameter values, the average of the stochastic approach follows the deterministic reaction, thereby conserving the substrate dependency of the flux in a stochastic manner. The applicability of Michaelis-Menten-kinetics to 2-dimensional case of membranes remains an open issue, but alternative formulations could be included easily in the modeling approach.

Here, the applicability of the hybrid, object-oriented approach has been demonstrated for the complex lipid metabolism in yeast. It now opens the way to more complicated types of analysis. For example, it will allow to understand the experimentally determined distributions of lipid species, fatty acids and saturation degrees. It also permits to investigate the effects of different types of perturbation and regulation such as gene knockout or overexpression or inhibition of specific enzymes.

The interesting challenge remains to extend the approach to the human or mammalian metabolism, which entails more different lipid species, fatty acid chain lengths and saturation degrees. The other way around, more detailed, time and compartment resolved measurements with newly available techniques would greatly advance the efforts in modeling yeast lipid metabolism.

## Author contributions

Conceived the work: EK, performed simulations: VS, JH, analyzed the data: VK, KT, JH, EK, wrote the paper: VS, KT, JH, EK. All authors read and approved the final version of the manuscript.

### Conflict of interest statement

The authors declare that the research was conducted in the absence of any commercial or financial relationships that could be construed as a potential conflict of interest.

## Supplementary material

Supplementary Table 1 **Model reactions**.

Supplementary Table 2 **Transport probabilities of all lipids to the different membranes**.

Supplementary Table 3 **Data Zinser et al. (1991) and model results**.

Supplementary Figure 1 **Comparison between classical deterministic and the model's stochastic approach to substrate dependencies applied to standard Michaelis-Menten kinetics with *V*_*max*_ = 100s^−1^ and Km = 30**.

Supplementary Figure 2 **Distribution of fatty acids**. The fraction of fatty acids in all simulated lipids in all membranes after one cell cycle compared. Blue bars represent model simulations (with error bars encoding the standard deviation of 1000 model simulations), the yellow bars the corresponding data from Martin et al. (2007).

Supplementary Figure 3 **Sensitivities**. Sensitivities of subcellular membrane composition to changes in *N*_*max*_. Colors represent changes in the range of 0.5% of the total lipid content of one membrane, larger values are printed in black (scaled by a factor of 100).

Supplementary Figure 4 **Membrane growth in the two test cases**. Model dynamics for the two test cases described in the main text. **(A,B)** Test case 1: Inhibition of ergosterol synthase. **(C,D)** Test case 2: Addition of inositol to the medium.
