# Supplementary figures and images for: Computational Modeling of Lipid Metabolism in Yeast
Source: Front Mol Biosci. 2016 Sep 27;3:57. doi: 10.3389/fmolb.2016.00057 (PMC5037213; doi:10.3389/fmolb.2016.00057)

Comparison between deterministic and stochastic approach

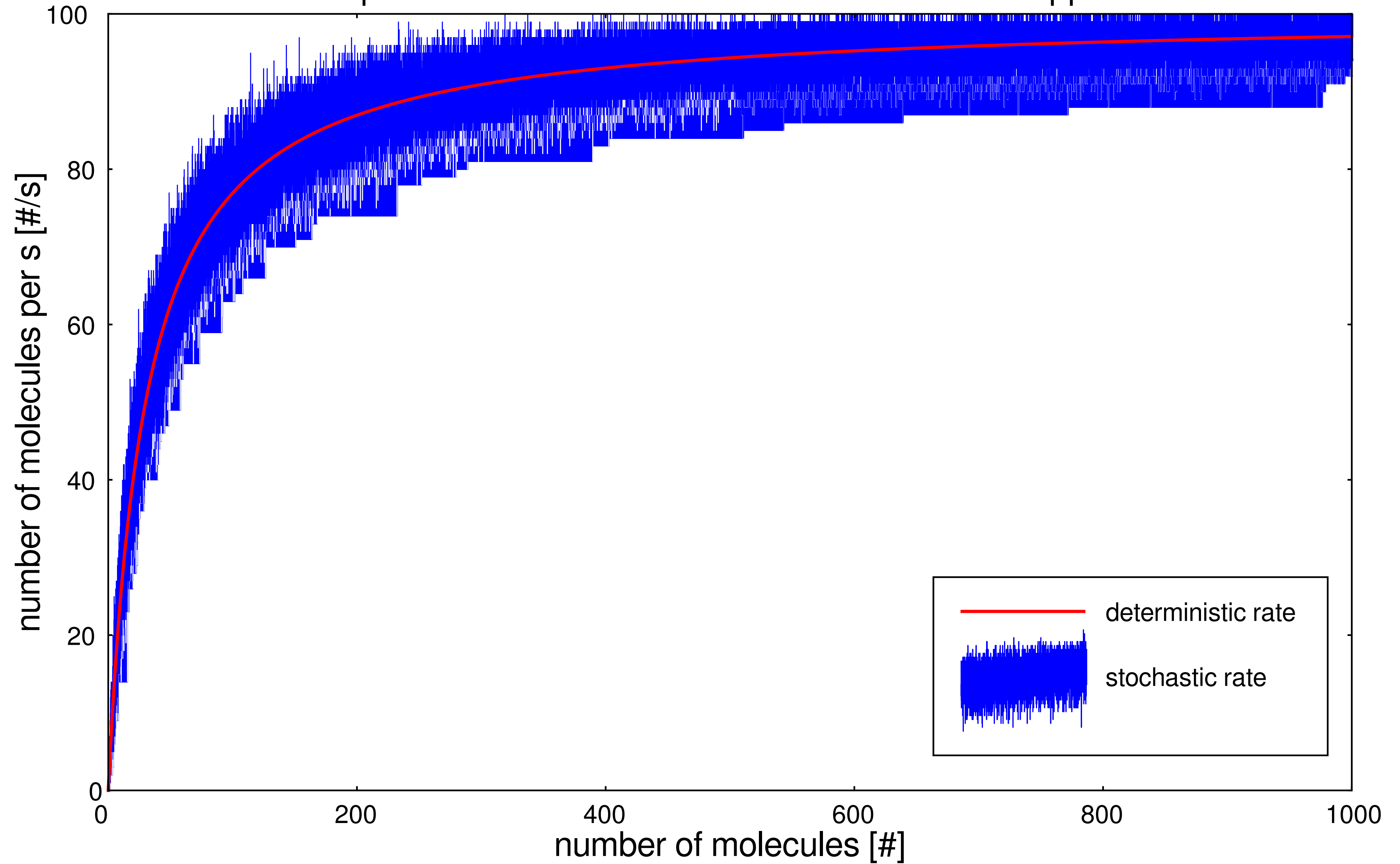

Supplement: Supplementary Figure 1 — Comparison between classical deterministic and the model's stochastic approach to substrate dependencies applied to standard Michaelis-Menten kinetics with Vmax = 100s−1 and Km = 30. [file Image1.pdf]

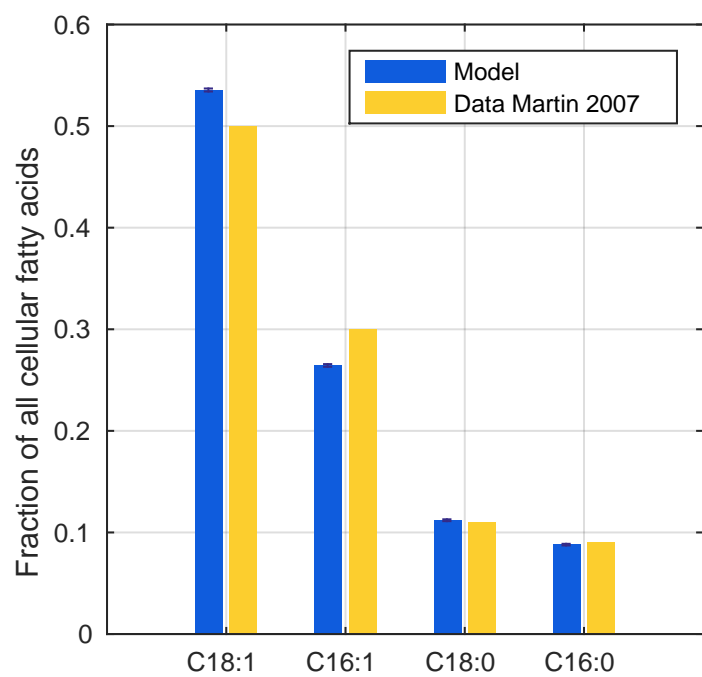

Supplement: Supplementary Figure 2 — Distribution of fatty acids. The fraction of fatty acids in all simulated lipids in all membranes after one cell cycle compared. Blue bars represent model simulations (with error bars encoding the standard deviation of 1000 model simulations), the yellow bars the corresponding data from Martin et al. (2007). [file Image2.pdf]

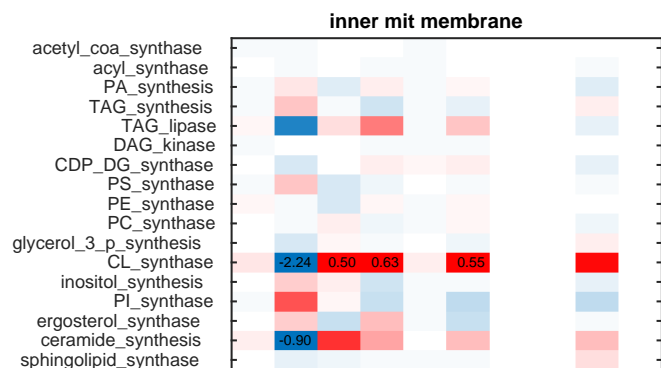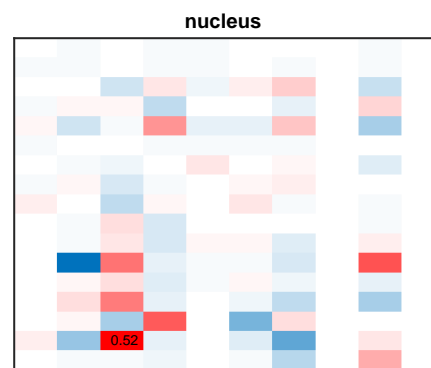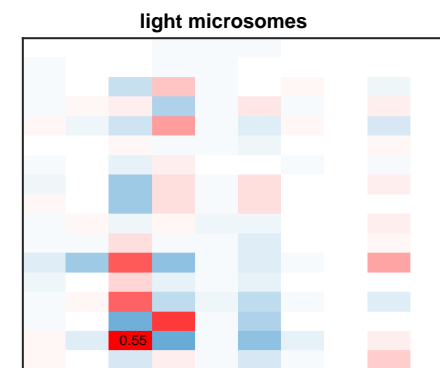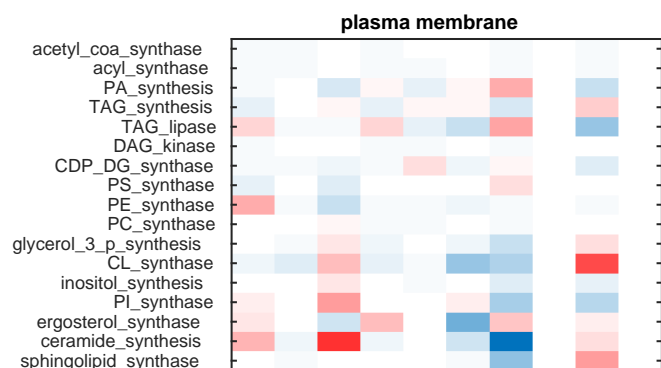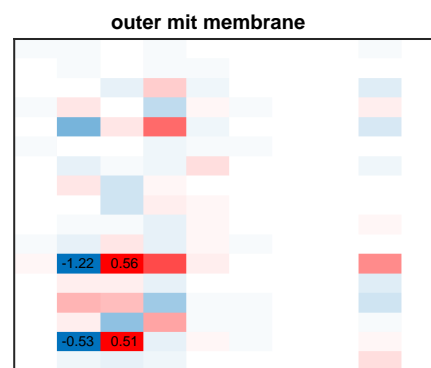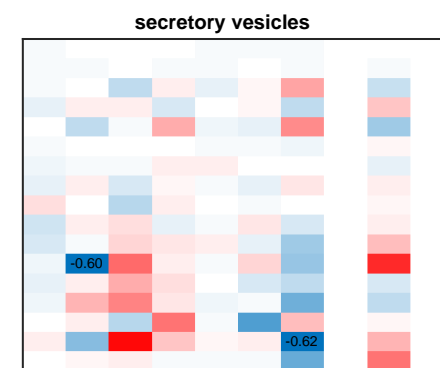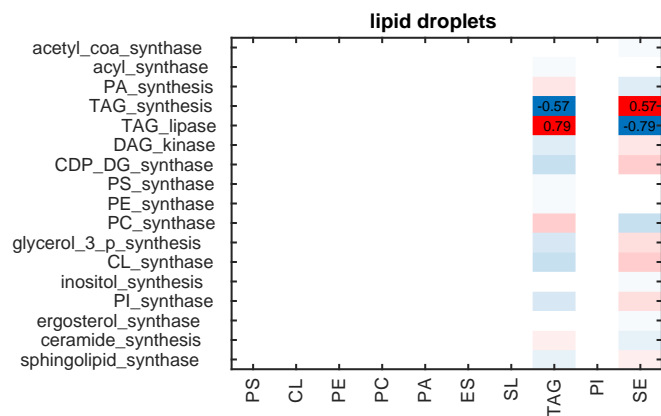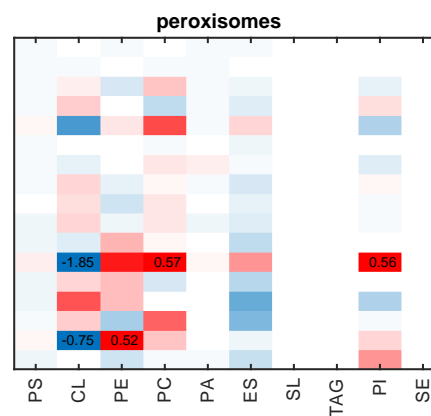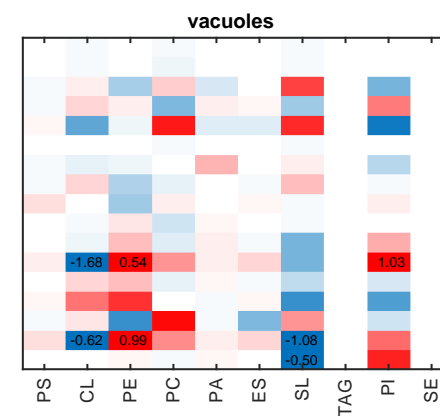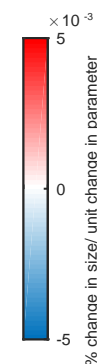

Supplement: Supplementary Figure 3 — Sensitivities. Sensitivities of subcellular membrane composition to changes in Nmax. Colors represent changes in the range of 0.5% of the total lipid content of one membrane, larger values are printed in black (scaled by a factor of 100). [file Image3.PDF]

# Terbinafine treatment

**A**

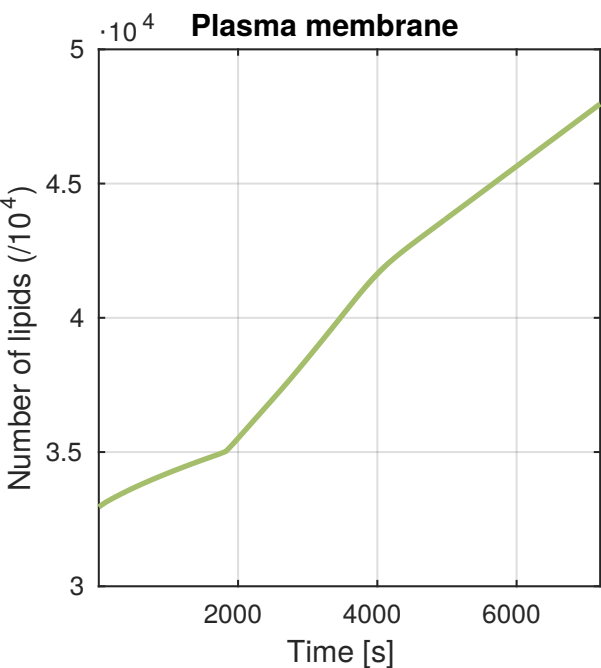

**B**

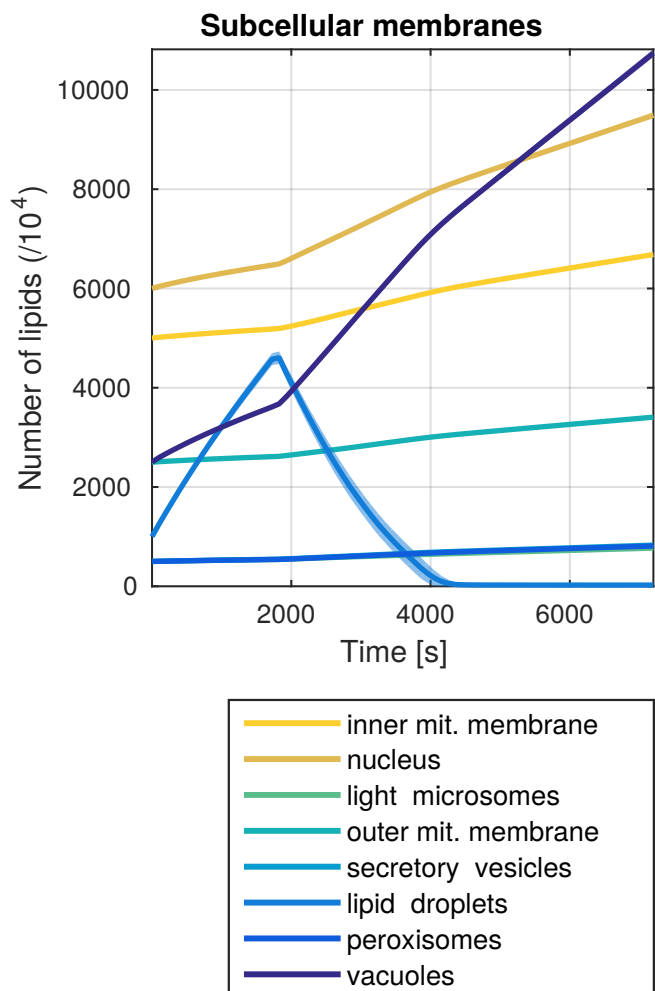

# Inositol addition

**C**

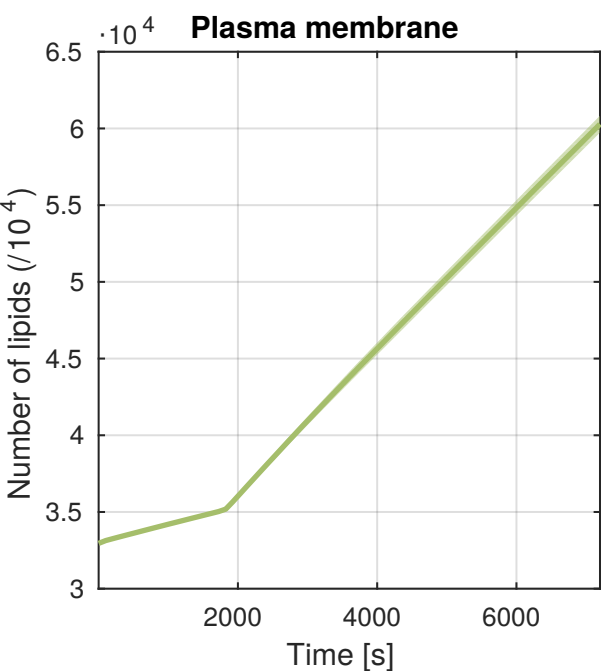

**D**

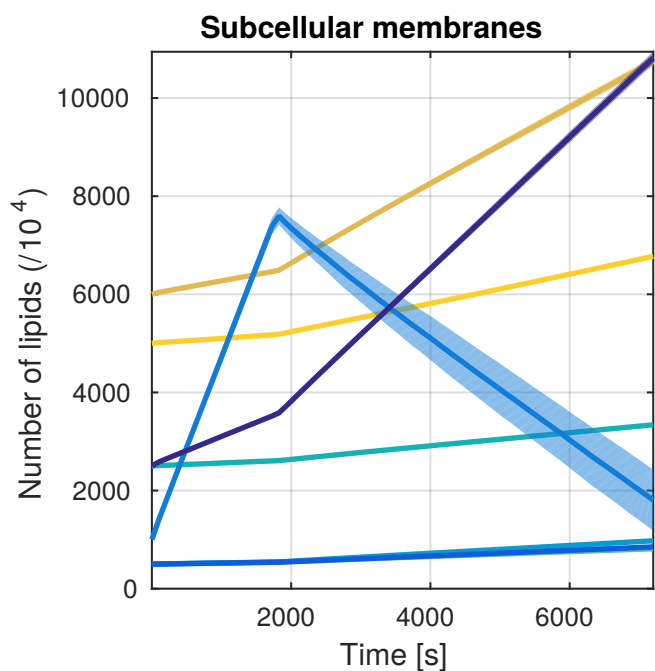

Supplement: Supplementary Figure 4 — Membrane growth in the two test cases. Model dynamics for the two test cases described in the main text. (A,B) Test case 1: Inhibition of ergosterol synthase. (C,D) Test case 2: Addition of inositol to the medium. [file Image4.PDF]
